# Is Living near Healthier Food Stores Associated with Better Food Intake in Regional Australia?

**DOI:** 10.3390/ijerph14080884

**Published:** 2017-08-07

**Authors:** Hamid Moayyed, Bridget Kelly, Xiaoqi Feng, Victoria Flood

**Affiliations:** 1 School of Health and Society, Faculty of Social Sciences, University of Wollongong, Wollongong, NSW 2522, Australia; xfeng@uow.edu.au; 2 Early Start Research Institute, Faculty of Social Sciences, University of Wollongong, Wollongong, NSW 2522, Australia; bkelly@uow.edu.au; 3 Menzies Centre for Health Policy, University of Sydney, Sydney, NSW 2006, Australia; 4 Faculty of Health Sciences, University of Sydney, Sydney, NSW 2006, Australia; vicki.flood@sydney.edu.au; 5 Westmead Hospital, Western Sydney Local Health District, Westmead, NSW 2145, Australia

**Keywords:** food environment, diet quality, food score, obesity

## Abstract

High prevalence of obesity and non-communicable diseases is a global public health problem, in which the quality of food environments is thought to play an important role. Current scientific evidence is not consistent regarding the impact of food environments on diet. The relationship between local food environments and diet quality was assessed across 10 Australian suburbs, using Australian-based indices devised to measure the two parameters. Data of dietary habits from the participants was gathered using a short questionnaire. The suburbs’ Food Environment Score (higher being healthier) was associated with higher consumption of fruit (χ^2^ (40, 230) = 58.8, *p* = 0.04), and vegetables (χ^2^ (40, 230) = 81.3, *p* = 0.03). The Food Environment Score identified a significant positive correlation with four of the diet scores: individual total diet score (r_s_ = 0.30, *p* < 0.01), fruit and vegetable score (r_s_ = 0.43, *p* < 0.01), sugary drink score (r_s_ = 0.13, *p* < 0.05), and discretionary food score (r_s_ = 0.15, *p* < 0.05). Moreover, the suburbs’ RFEI (Retail Food Environment Index, higher being unhealthier) showed a significant association with higher consumption of salty snacks (χ^2^ (24, 230) = 43.9, *p* = 0.04). Food environments dominated by food outlets considered as ‘healthier’ were associated with healthier population food intakes, as indicated by a higher consumption of fruit, vegetables, and water, as well as a lower consumption of junk food, salty snacks, and sugary drinks. This association suggests that healthier diet quality is associated with healthier food environments in regional Australia.

## 1. Introduction

High prevalence of obesity and non-communicable diseases (NCDs) is a global public health problem, and the food people eat is a major contributing factor [1]. A variety of factors contribute to obesity and NCDs [1], and many public health and nutrition experts and policymakers believe that food environments play an important role [2]. Potentially important aspects of food environments that shape obesity and NCD risk have been conceptualised across a variety of settings, such as the home and wider community [3]. The community includes schools, workplaces, and public transport facilities, amongst others [2]. However, it remains to be assessed whether different types of vendors within any food environment have a positive or negative effect on health [4].

Current scientific evidence is not consistent regarding the impact of food environments on diet. Some studies have identified positive associations between unhealthy diet and/or weight and the food outlet composition of food environments [5,6,7,8,9]. There are many studies that have reported a positive association between unhealthier food consumption, unfavourable health outcomes, and certain food environments such as those dominated by fast food restaurants, takeaways, and small convenience shops with fewer food choices, hence such food outlet types are often labelled as “unhealthier” [10,11,12]. On the other hand, certain food environments described by the dominance of food store types, such as supermarkets, groceries, and restaurants, have been associated with healthier diets, eating behaviours, and decreased rates of overweight and obesity [13]. However, studies have also shown the opposite relationship or null findings [11]. Furthermore, the association between living in a deprived food environment and having increased access to unhealthier food outlet types is not consistently observed in other countries apart from the United States (US) [14]. This inconsistency may, in part, be due to variations in food environments across countries, differences in food cultures and traditions, and also the different ways in which diet and the food environment have been measured across studies [15]. Hence, it is difficult to ascertain the extent to which dominance of a particular food outlet type would contribute to the healthiness or unhealthiness of that particular food environment across the globe.

Little data is available on the nature and healthiness of food environments outside of the US, including in the Australian context. As there may be differences between the food environment of the US and other countries [16], the results of US-based studies are not directly or universally applicable [14]. Variations in food environments across countries may relate to the geographic size of ‘urban’ food environments and the residential distribution of socioeconomic and ethnic groups [17]. The aim of this research was to explore the association between food environments and diet quality among people living in a regional area in Australia.

## 2. Materials and Methods

We assessed the relationship between local food environments and diet quality across 10 Australian suburbs, developing Australian-based indices of the two parameters. Food environment indices were taken from our previous studies [18] and diet quality indices were newly devised, aiming to develop a short tool that can be easily applicable across large populations in future Australian studies. Our study implemented a short questionnaire with the aim to understand the dietary habits of residents of several Australian suburbs using short diet questions, as well as to correlate this to the quality of the food environment in which they lived. The Australian Guide to Healthy Eating food categories and recommendations [19] were used to develop a scoring system to assess diet quality.

### 2.1. Sampling

The sampling frame consisted of 2000 households across 10 suburbs of the Illawarra region of New South Wales, Australia. For each suburb, a central point was created of a polygon (a closed segment of area under study) made of the “main shopping area” of all the food outlets in each suburb using geographic information system software ArcGIS. The actual size of the final polygon varied depending on the housing density in each suburb. Households were selected from those closest to the central point first (distance measured by walkable road network), with a limit of 200 households per suburb and within a buffer of 2 km from the main shopping area. Two thousand envelopes containing a questionnaire, participant information sheet, and a reply-envelope were delivered by two researchers to households’ mailboxes in all selected suburbs during July and August 2014. The address of each household was recorded and each questionnaire was given a unique code. The person with the main or shared responsibility for grocery purchases in each household was asked to complete the questionnaire and mail it back to the investigators main office within two weeks. No incentives were available for participation. The study received ethics approval from the University of Wollongong Human Research Ethics Committee. Ethics Approval Project Id Code is “HE14/208”.

#### 2.1.1. Assessment of Food Outlets in Suburbs

Data on the types, number, and location of food outlets in 23 suburbs of the Illawarra region surrounding Wollongong were obtained by direct observation in an earlier study [18]. All the food outlets in each suburb were geocoded using ArcGIS and classified by store type using a tool based on other research [20,21] and the outcomes of a Delphi survey with 31 health and nutrition experts using two rounds of questionnaires [22]. From this process, 24 distinct store types found in Australian suburbs were classified along a 20-point scale as healthy or unhealthy (from +10 healthiest to −10 unhealthiest). Thus, for this study, all the stores in the 23 suburbs were given a score based on their type (e.g., fruiterer/green grocer +10, supermarket +5, fast food franchise −10), likewise for all food outlet types. Within each suburb, all available food outlets’ scores were then summed. A suburb’s Food Environment Score (FES) was devised as the sum of the healthiness ratings for all available food outlets in each suburb divided by the total number of food outlets in that particular suburb. A higher score represents a “healthier” food environment. From these 23 suburbs, the five suburbs with the highest total healthiness scores and the five suburbs with the lowest total healthiness scores were selected for this study.

The FES was also compared to another food environment-related score available from the literature: the Retail Food Environment Index (RFEI) [23]. The RFEI was originally devised to assess the relationship between the availability of different types of food retailers (including fast-food restaurants, convenience stores, grocery stores, supermarkets) and obesity among adults in suburbs across a region of Canada (Spence et al., 2009) [23]. The RFEI is calculated as the ratio of “fast-food restaurants (F) and convenience stores (C)” within a given radius (including takeaways, pizzerias, convenience stores, gasoline stations with convenience stores, and convenience neighbourhood stores that also sell selected grocery items, but not restaurants) to “grocery stores and produce vendors (G) including supermarkets, ethnic stores, and upscale organic markets” in an area, using formula (F + C)/G, with a higher score indicating a less healthy food environment.

#### 2.1.2. Assessment of Food Intake

A short questionnaire was developed to obtain information on the dietary intake of the participants. Eleven questions asked about intakes of servings of fruit and vegetables, and frequency of discretionary food (fast food, junk food, salty snacks) and cups of sugary drinks (soft drinks, energy drinks, fruit juices), type of milk usually consumed, and amount of water consumed per day. These diet questions were based on the New South Wales (NSW) Population Health Survey, based on recommendations by [24]. Most of these questions have been validated in a range of sub-populations, with a particular focus on children and adolescents, but with less information in adult populations.

#### 2.1.3. Calculation of the Diet Scores

Food types were grouped together to create four diet score components: (1) fruit and vegetable score, (2) discretionary food score, (3) sugary drink score, and (4) water score, with a final addition of each component score to derive an individual’s Total Diet Score. Food frequency responses were dichotomised (0 = less healthy food choices or 1 = healthy food choices), with cut-points given based on nutrition guidelines for each food/drink group using the Australian Guide to Healthy Eating [19] recommended servings, where available and as suitable for short questions. The following cut-points were used (see Table 1):

1. A score of 1 point was given for consumption of healthy choices in each food category: fruit > 2 servings/day; vegetables > 4 servings/day; take away foods < 1/week; French fries < 1/week; salty potato crisps < 1/week; fruit juice < 1/week; soft drinks < 1/week; energy drinks < 1 week; and water > 3/day
2. A score of 0 points was given for all other responses

The scoring system allowed a maximum possible score of 9 through 3 points from “consuming enough of healthful food items” (fruit, vegetables and water) and 6 points from “consuming less of unhealthful food items” (Table 1). In addition to using the questions in an index score, each question was assessed individually in relation to the FES and the RFEI.

#### 2.1.4. Analysis

Data were analysed using SPSS for Windows version 19.0 (SPSS Inc., Chicago, IL, USA). ArcGIS version 9.3 (ESRI Inc., Redlands, CA, USA) was used to map and geocode the food outlets. The outlets were plotted using their co-ordinates that were “ground-truthed” in an earlier study [18]. The Spearman rho correlation coefficient for ordinal data was calculated for the relationship between suburbs’ FE score and each of the diet scores, as it measures the strength of the association between two ranked variables, showing how closely the two sets of data are linked. Pearson chi-square test of statistical significance was calculated for the relationship between each suburb’s FES dichotomised into <median FES and >median FES and dietary choices of individuals as indicated by their questionnaire responses, and similarly also using the RFEI dichotomised into <median RFEI scores and >median RFEI scores and participants’ dietary choices.

## 3. Results

Overall, 230 households returned a completed questionnaire (response rate 11.5%). These included 167 (73%) females and 63 (27%) males. One-third of the participants were less than 50 years old (*n* = 76) and two-thirds (*n* = 154) were 50 years old or more. Most participants were female (*n* = 167). More participants belonged to FES > median (*n* = 137), that is, more people lived in areas with better food environments. Suburbs with higher healthiness scores had a higher (overall 12%) participation rate compared with suburbs with lower healthiness scores (overall 8%).

### 3.1. Relationship between Food Environment Score and Individuals’ Diet Index Scores

There was a medium to weak positive correlation between a suburb’s Food Environment Score and its residents’ various diet scores, i.e., individual total diet score (r_s_ = 0.30, *p* < 0.01), fruit and vegetable score (r_s_ = 0.43, *p* < 0.01), sugary drink score (r_s_ = 0.13, *p* < 0.05), and discretionary food score (r_s_ = 0.15, *p* < 0.05). The Food Environment Score, where higher scores indicate healthier food stores, identified a significant positive correlation with four of the diet scores.

### 3.2. Association between Food Environment Score, RFEI, and Consumption of Fruit and Vegetables

Firstly, chi-square tests of independence were performed to examine the relationship between the suburbs’ FES and dietary consumption of fruit and vegetables. There was a significant association between FES and fruit consumption (χ^2^ (40, 230) = 58.8, *p* = 0.04), as well as FES and vegetable consumption (χ^2^ (40, 230) = 81.3, *p* = 0.03). Pearson chi-square tests were calculated for the relationship between each suburb’s food environment score and also RFEI, each dichotomised into <median scores and >median scores, and the dietary choices of individuals as indicated by their questionnaire responses, classified into healthier and less healthy categories. A higher proportion of people reported consuming 2 or more servings of fruit per day or 5 or more servings of vegetables per day if they lived in an area with an FES greater than the median compared to those people who lived in an area with an FES less than the median (see Table 2). No other associations between the consumption of individual food types and FES were identified.

### 3.3. Association between Food Environment Score, RFEI, and Consumption of Individual Food Categories

Using the Chi square test, the following shows the summary of findings in relationships between the FE score, the RFEI, and the consumption of individual food types (diet categories Fruit and Vegetables/Salty snacks/Sugary drinks/Energy drinks/Water) (See Table 3):

## 4. Discussion

The outcomes of our analyses demonstrate that healthier food environments are associated with healthier food intakes, as indicated by the higher consumption of fruit and vegetables, and the lower consumption of unhealthier food types including junk food, salty snacks, and sugary drinks. A higher availability of unhealthier food outlet types was associated with unhealthier food intakes. The aim of our study was to quantify food environments, develop a diet quality scoring system, and assess the relationship between the two parameters in the Australian context. In this study, the suburbs’ FES was positively correlated with several diet quality scores. The FES (higher being healthier) was associated with a higher consumption of fruit and vegetables. Our developed scoring system for the FE of suburbs seems to be an indicator of healthiness of diet consumed by the individuals residing in those suburbs. This is because our suburb-level FES was highly correlated with some diet quality scores of participants.

One significant association of our “healthy” FES was with a higher consumption of fruit and vegetables among individuals. This has been mentioned by several studies in the available literature that found a positive relationship between the food environment and dietary quality. Izumi et al. (2011) used Food Frequency questionnaires to evaluate the relationship between directly observed neighbourhood availability and the consumption of dark-green and orange vegetables. The results showed 0.17 more daily servings of vegetables for residents of areas with more opportunities for buying these foods compared to residents of areas with no neighbourhood food outlets selling these foods [25]. In a study by Morland et al. (2002), the fruit and vegetable intake of the studied population increased by 11 to 32% for each additional supermarket geocoded in the census tract for the study population [26]. In another study that used the GIS method, Bodor et al. (2008) found that the availability of small food outlets within 100 m of residences was significantly associated with higher mean intakes of fruit and vegetables (*p* = 0.083) [6]. Caldwell et al. (2008) used a participant survey to evaluate the relationship between accessibility to fruit and vegetables in the community and change in fruit and vegetable intake in a community-based health promotion program in Colorado, US, and found that greater accessibility to fruit and vegetable vendors was associated with increased consumption [9]. Neighbourhoods’ supermarket density has also been linked with a healthy diet quality of residents [27]. In another study, Lucan and Mitra (2012) found a greater consumption of fast food in food environments with poor availability and accessibility to supermarkets [28]. In a study by Michimi and Wimberly (2010), food diaries were used to examine the relationship between travel distance to supermarkets and obesity and fruit and vegetable intake in adults, showing a positive association between distance to supermarkets and obesity [29]. Supermarkets, fruiterers, and green grocers are placed among healthier food outlets types in our system of food outlet scoring. Hence, our suburb-level food environment scores seem to be indicators of the healthiness of diets consumed by the individuals residing in those suburbs.

As mentioned, RFEI measured for Australian suburbs in our study was associated with a higher consumption of salty snacks, and several studies have also identified this association in the literature. Based on our findings, RFEI can be used as an indicator to identify poor food environments, and individuals living in poorer food environments may have poorer food intake, at least for some food items. Our tool has classified fast food outlets and convenience stores into unhealthier food outlet types, among others. Jeffery et al. (2007) reported a significant inverse association between BMI and the number of fast food outlets within a 2-mile radius of home addresses of participants, also making use of GIS for investigating this relationship [7]. A study in Australia found a negative relationship between the availability of fast food and convenience stores close to homes, and consumption of fruit and vegetables in children [30]. A study by Laska et al. (2010) showed a positive relationship between BMI and the availability of a convenience store within a 1600-m buffer of households [8]. In a study by Burgoine et al. (2014), food frequency questionnaires were used to measure dietary quality and the relationship with food environments. The results showed that exposure to takeaway food outlets were positively associated with the consumption of takeaway food [31].

Most previous studies have only focused on assessing the availability or density of a limited number of food outlet types, while our study incorporated all available food outlet types within the scoring system. Another strength of this study is the classification of food outlet types as healthier or less healthy based on the Delphi method of expert consensus, as explained elsewhere [22], which is an important consideration in attempting to evaluate the overall nature of the food environment.

One limitation in the development of the total diet score is that it assumes equal weighting of the component scores which contribute to the total diet score, i.e., fruit and vegetable score, junk food score, sugary drink score, and water score, which add up to form the total diet score for an individual. Further, there are 3 points from “consumption of positive food” items (fruit, vegetables, and water) and 6 points from “not consuming negative food” items. This scoring system needs further validation as currently it is merely a reflection of the questions included in the short survey. It is acknowledged that although there is no validity of the food environment score index, which represents an opportunity for future work. This study partly contributes to convergent validity assessment, by testing to see if the FE score is correlated with those diet factors that are thought to be linked with FE. Additionally, the assessment of diet was based on simple short questions, and although we have some validity information about these short questions, in particular among children and adolescents [24], there is limited information about their use in adult population groups. We know from previous validity work that people tend to overreport their ‘good’ foods and underreport ‘bad’ foods, and so caution is required in the interpretation of the food people report using such short questions. This may limit our ability to identify an association between some food types and the food environment.

The sample size of the participants was small (*n* = 230), and the number of suburbs considered is also limited (*n* = 10). The response rate for our mailed questionnaire was 11.5%, however, response rates to published mail surveys tend to be low [32]. Lack of incentives [33], the nature of the questionnaire [34], and, as in our case, the length of the questionnaire [35] can attribute to such low response rates in questionnaire surveys mailed to residential populations. Although the number of studied suburbs was limited, the tool of food outlet types is built upon work by other researchers as well as on our earlier pilot studies that have incorporated all the food outlet types available across a larger geographical area in Australia. The amount of data collected, especially by walking and ground-truthing each shop, geocoding the locations into ArcGIS software, verifying the food outlet types, categorising them appropriately by the tool, and doing so across 10 suburbs, was a challenging task. Further polygons of each suburb were targeted to be close to the shopping areas, and 2000 participants were handed over envelopes in their mailbox by walking to them, of which 230 responded, thus amounting to a richness of data and groundwork, which is a strength of this study.

Another limitation is that the study was conducted in July–August (winter in the southern hemisphere), and dietary data may be impacted by seasonal variation, such as lower soft drink intakes in colder weather. Another limitation, as the questionnaire is addressed to households, is that it might be possible for the participants to discount or underrate the foods they consume at workplaces, education sites, or during travel and transport, e.g., use of vending machines [35]. This again can affect the healthfulness of food consumption scores. Furthermore, socioeconomic status has a significant impact on individuals’ dietary behaviours. Low Socio economic status (SES) may place adults at risk for poorer health situations for a range of reasons, such as difficult living conditions, less access to healthcare, more access to unhealthy food options, more psychological stress [36], engaging in risky health behaviours [37], and reduced availability of fresh fruits and vegetables [38]. In this study, SES measures were not included; this is considered as a limitation, however, the association between indicators of SES and food environment has been addressed by the researchers in a separate study.

Community food environment indicators have been used to measure and monitor the number, type, location, and density of different food outlets in residential areas and have been compared to health outcomes [3,4,15]. Consumer food environment indicators include various and specific food products’ availability, prominence (including information and promotions as well as the store space that they comprise), and accessibility (including price affordability and ease of locating items within stores with respect to individual consumers) [3,4,15]. Since the food premise/outlet type is not an appropriate proxy for dietary contents available inside the premise, it should be added that our study, through the FES, has focused on studying community food environment and perhaps from the aspect of consumer food environment, further research can be conducted to correlate the healthiness of each food outlet type with the healthiness of the dietary contents available at the food outlet type.

Further scope for analyses could be to include socioeconomic and demographic parameters of each suburb (e.g., education, income), and to relate such data with the accessibility of food outlets in the suburbs. The proposed scoring and classification system of food outlet types can be applied over a larger geographic area, and data from larger FFQs or diet questionnaire studies can be used to explore and compare the relative healthiness and diet quality of community food environments, as well as to identify places in need of improvements. A diet quality index, such as ours, is considered to be a useful tool for public health practitioners in assessing studied participants’ adherence to current dietary recommendations [39] and in evaluating and correlating diet quality with other parameters.

Valid and reliable measures of food environments can inform both research and action in terms of designing interventions and policy change towards improving the healthiness of these environments [2]. Such changes may then have an impact on diet quality and health. Food environment measures need to be applicable across a variety of areas with different sociodemographic and cultural distributions so that actions can be identified and applied to areas most at-risk. Since this study was a cross-sectional analysis of a small dataset, a causal relationship cannot be concluded. After further validation of our scoring system, it can be recommended to include analyses of suburbs for ‘healthiness’ of their food environments, since food environments are found to be linked with the diet quality of the people. This can potentially be achieved through the application of practicable tools across Australian suburbs, including a variety of regional and urban areas.

## 5. Conclusions

This study aimed to assess the diet quality of people living in 10 suburbs in Illawarra region of New South Wales, Australia, to demonstrate the relationship between the healthiness of neighbourhood food environments and the dietary intake of residents. Of the studies relating the dietary quality and food environments in Australia, this work is among the first to measure the relative healthiness of food environment and diet quality. Our suburb-level food environment scores seem to be good indicators of healthiness of diet consumed by the individuals residing in those suburbs. Further scope for analyses include socioeconomic, demographic, and accessibility parameters of suburbs. Applying such tools over data of food outlets and diet quality across larger geographic areas can help researchers to explore relationships between food environments and diet quality to further understand Australian food environments.

## Figures and Tables

**Table 1:** Questionnaire questions and scores regarding diet intake of participants.

Question as in the Questionnaire:	Score Categorisation
How many servings of fruit do you usually eat each day? Include all fresh, dried, frozen, tinned (servings defined)	1 serving or less/day = 0
	2 or more servings/day = 1
How many servings of vegetables do you usually eat each day? (servings defined)	Less than 4 servings per day = 0
	4 or more servings = 1
**Total of the above 2 =**	**Fruit and Vegetable Score**
How often do you have meals or snacks such as burgers, pizza, and chicken from take-away places?	Less than once a week = 1
	All options indicating once a week or more = 0
How often do you eat hot chips, French fries, wedges, or fried potatoes?	Less than once a week = 1
	All options indicating once a week or more = 0
How often do you eat potato crisps, or other salty snacks? Include snacks such as twisties or corn chips	Less than once a week = 1
	All options indicating once a week or more = 0
**Total of the above 3 =**	**Discretionary Food Score**
How many cups of fruit juice do you usually drink?	Less than one cup a week = 1
	All options indicating once a week or more = 0
How many cups of soft drink, sports drink, or cordial do you drink?	I never have these drinks = 1
	1 to 3 cups a week or less = 0; More than that = 0
How many cups of energy drinks do you drink?	I never have these drinks = 1; All other options = 0
**Total of the above 3 =**	**Sugary Drink Score**
How many cups of water do you usually drink?	Less than 3 cups a day = 0; 3 cups or more a day = 1
**Based on the above Question =**	**Water Score**
(a) Fruit and vegetable score: The maximum score for each individual could be 2 and the minimum 0. A higher score indicates a higher consumption of fruits and vegetables by a participant. (b) Discretionary food score: The maximum score for each individual could be 3 and the minimum 0. A higher score indicates a lower consumption of such foods by a participant. (c) Sugary drink score: The maximum score for each individual could be 3 and the minimum 0. A higher score indicates a lower consumption of sugary drinks by a participant. (d) Water score: If participant selected recommended portion of water, score is 1, if not it is 0. (e) Total diet score: Individuals’ total diet score generated by adding the score for each diet component.	

**Table 2:** Association of FES (Food Environment Score), RFEI (Retail Food Environment Index), and food consumption.

	FES < Median (%)	FES ≥ Median (%)	RFEI < Median (%)	RFEI ≥ Median (%)
Fruit				
2+ servings/day	64.9% ^1^	86.8% ^1^	84.4%	76.9%
Vegetables				
4+ servings/day	6.4% ^1^	25.7% ^1^	58.3%	48.5%
Takeaway				
<1 serving/week	83%	85.3%	83.3%	87.3%
French fries				
<1 serving/week	74.5%	83.8%	78.1%	83.65
Salty snacks				
<1 serving/week	76.6%	84.6%	84.4%	80.6%
Fruit juice				
<1 serving/week	73.4%	79.4%	81.3%	73.9%
Soft drink				
“never have”	44.7%	58.8%	89.6%	88.8%
Energy drink				
“never have”	93.6%	98.5%	95.8%	97%

^1^ *p* values <0.05 using Pearson chi-square test, other results were statistically not significant.

**Table 3:** Association between Food Environment Score, RFEI, and Consumption of Individual Food Categories.

Food Environment Score Relationship with:	Following Results with *p* Values ≤ 0.05
Fruit consumption	χ^2^ (40, *n* = 230) = 58.8
Vegetable consumption	χ^2^ (40, *n* = 230) = 81.3
**RFEI relationship with:**	
Salty snack consumption	χ^2^ (24, *n* = 230) = 43.9
